# A305 ANONYMOUS LIVING LIVER DONATION IMPROVES ACCESS FOR MEDICALLY UNDERSERVED CHILDREN IN NEED OF LIVER TRANSPLANTATION

**DOI:** 10.1093/jcag/gwad061.305

**Published:** 2024-02-14

**Authors:** T Yodoshi, T John, J Stunguris, M De Angelis, K Van Roestel, J Hensley, Y Avitzur, R Bandsma, N Jones, B M Kamath, S Ling, M Miserachs, A Ghanekar, M Cattral, B Sayed, V L Ng

**Affiliations:** The Hospital for Sick Children, Toronto, ON, Canada; The Hospital for Sick Children, Toronto, ON, Canada; The Hospital for Sick Children, Toronto, ON, Canada; The Hospital for Sick Children, Toronto, ON, Canada; The Hospital for Sick Children, Toronto, ON, Canada; The Hospital for Sick Children, Toronto, ON, Canada; The Hospital for Sick Children, Toronto, ON, Canada; The Hospital for Sick Children, Toronto, ON, Canada; The Hospital for Sick Children, Toronto, ON, Canada; The Hospital for Sick Children, Toronto, ON, Canada; The Hospital for Sick Children, Toronto, ON, Canada; The Hospital for Sick Children, Toronto, ON, Canada; University Health Network, Toronto, ON, Canada; University Health Network, Toronto, ON, Canada; University Health Network, Toronto, ON, Canada; The Hospital for Sick Children, Toronto, ON, Canada

## Abstract

**Background:**

Since our first pediatric anonymous non-directed live donor liver transplant (A-LDLT) performed in April 2005, 62 children have undergone live donor liver transplant (LDLT) with an anonymous non-directed graft. Anon-LDLT organs being allocated as per our deceased donor liver transplant (DDLT) wait list.

**Aims:**

To evaluate clinical outcomes, recipient characteristics and social determinants of health of pediatric recipients of A-LDLT in comparison to those who received a directed LDLT (Dir-LDLT) and DDLT.

**Methods:**

Retrospective analysis of all recipients of LDLT performed between January 2005 and March 2023. Demographic and clinical data included age, sex, race, ethnicity, single-parent households, primary diagnosis, recipient blood type, time on waiting list, post-LT intensive care unit (ICU) length of stay (LOS), time to extubation, and post-transplant comorbidities were assessed as covariates. A comparative analysis was conducted between children receiving A-LDLT, Dir-LDLT versus DDLT.

**Results:**

A total of 422 (50% male, 57% white, 23% Asian, 7% Black, 5% indigenous) childrenunderwent LT during the study period. Biliary atresia (40%) and metabolic diseases (19%) were the commonest primary indications. A-LDLT was done in 62 children (15%) with no directed donor options. Dir-LDLT was in 174 children (41%). Of 186 DDLTs (44%), 25% were split grafts, 26% reduced, and 49% whole liver grafts. In A-LDLT recipients, the primary diseases were more frequently characterized by metabolic disorders (31%), while the incidence of acute liver failure was notably lower (0%). Recipients of Anon-LDLT were more often Indigenous recipients in comparison to Dir-LDLT or DDLT recipients (p=0.044). Anon-LDLT recipients were more frequently living in single parent households (18% vs 3% vs 10%, pampersand:003C0.001) and to require interpreter assistance (11% vs 3% vs 0.5%, pampersand:003C0.001) (**Table**), compared to children who received Dir-LDLT or DDLT. Median time on the wait list was longer for Anon-LDLT (92 days) compared to Dir-LDLT (62 days) and DDLT (72 days) recipients (p=0.004). Post-LT ICU LOS, time to extubation, other post-LT complications or patient prognosis were better in the A-LDLT and Dir-LDLT recipients than DDLT recipients.

**Conclusions:**

This retrospective analysis of 422 children undergoing LT at a single institution confirms excellent patient and graft survival for A-LDLT recipients. Patients from Indigenous communities, single parent and households where English is a second language, are more frequently the beneficiaries of Anon-LDLT grafts. Anon-LDLT, as utilized in this single center analysis, benefits medically underserved pediatric patients who otherwise have limited access to the advantages of LT.

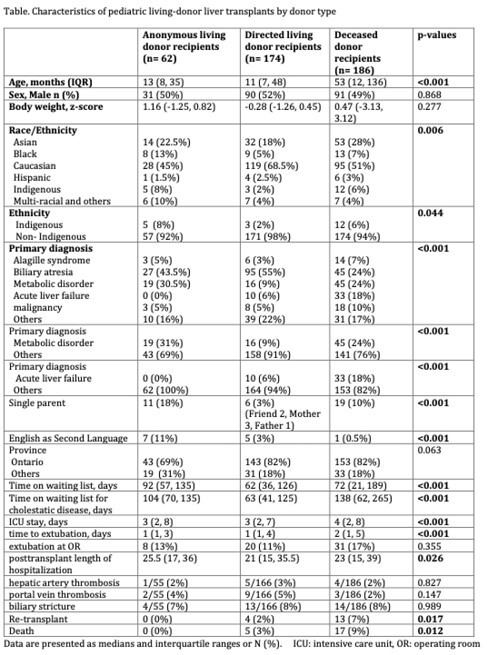

**Funding Agencies:**

None

